# Cholecystitis and Liver Abscess Caused by Oral Bacteria

**DOI:** 10.7759/cureus.108280

**Published:** 2026-05-05

**Authors:** Ryosuke Kikuchi, Tomohiro Suzuki, Yutaka Ejiri, Mayumi Tai, Ichii Osamu

**Affiliations:** 1 Gastroenterology, Fukushima Rosai Hospital, Iwaki, JPN

**Keywords:** actinomyces naeslundii, cholecystitis, liver abscess, oral bacteria, porphyromonas gingivalis

## Abstract

The concept that oral foci of infection can have systemic effects is known as “oral focal infection.” Here, we report two rare cases of hepatobiliary infections caused by oral bacteria: *Porphyromonas gingivalis* and *Actinomyces naeslundii*. Case 1 involved acute cholecystitis, whereas Case 2 presented with a liver abscess. Both patients had poor oral hygiene, suggesting hematogenous spread from the oral cavity. These cases underscore the potential for oral pathogens to cause systemic infections and highlight the importance of oral hygiene and interprofessional collaboration between medicine and dentistry for prevention.

## Introduction

The concept that oral foci of infection can have systemic effects is known as “oral focal infection.” This concept has gained significant attention alongside the growing global emphasis on oral hygiene. Infective endocarditis caused by the colonization of oral bacteria on the endocardium is a representative example of this concept [1]. However, the primary pathogens in hepatobiliary infections, such as cholecystitis and liver abscess, are gut-derived bacteria, including *Escherichia coli*, *Klebsiella spp.*, and *Enterococcus spp.* [2,3]. And it is rare for oral bacteria to be the causative agent. In this report, we present two cases of hepatobiliary infections caused by *Porphyromonas gingivalis* and *Actinomyces naeslundii*. Both cases were characterized by poor oral hygiene, and these specific bacteria are closely associated with the pathogenesis of periodontal disease.

## Case presentation

Case 1

The patient was a 70-year-old man, hospitalized for rehabilitation following a cerebral infarction, who developed vomiting, abdominal distension, and fever. Blood tests revealed liver dysfunction, and computed tomography (CT) imaging showed gallbladder enlargement, raising suspicion of acute cholecystitis. The patient was transferred to our hospital for management. His medical history included cerebral infarction, type two diabetes mellitus, atrial fibrillation, and hypertension. He had no history of alcohol consumption, smoking, or a family history of liver disease.

Upon admission, the patient was alert and afebrile. Jaundice was not present. He exhibited global aphasia and right hemiplegia due to the cerebral infarction, with manual muscle testing scores of 1. Abdominal examination revealed mild distension and tenderness in the right upper quadrant. Murphy’s sign was not clearly evident. His activities of daily living were severely limited, requiring full assistance with feeding and diaper use.

**Figure 1:**
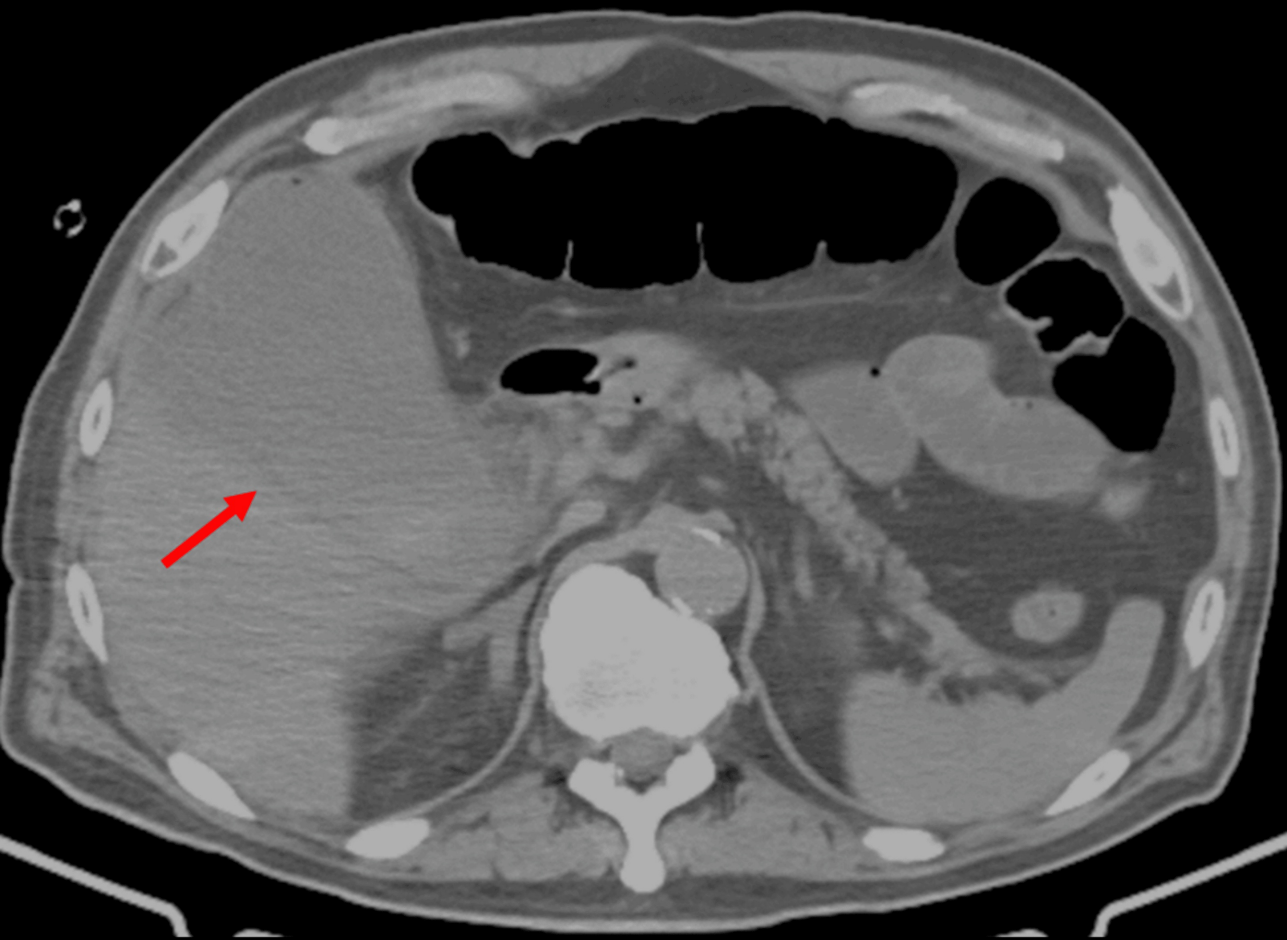
Unenhanced abdominal CT shows a gallbladder lesions. The red arrow points to an enlarged gallbladder. An abscess is noted on the liver surface, contiguous with the gallbladder.

Laboratory tests showed leukocytosis and elevations in C-reactive protein (CRP), aspartate aminotransferase (AST), and alanine aminotransferase (ALT) (Table 1). Inflammatory markers were significantly increased. Blood glucose was 189 mg/dL, and HbA1c was 6.0% (Table 1). Glycemic control was well-maintained. Unenhanced abdominal CT revealed an enlarged gallbladder and a subcapsular hepatic abscess contiguous with the gallbladder but no gallstones (Figure 1). The patient was diagnosed with acute cholecystitis.

**Table 1:** Summary of clinical, laboratory, and imaging findings in two cases Abbreviations: WBC, white blood cell count; CRP, C-reactive protein; AST, aspartate aminotransferase; ALT, alanine aminotransferase; ALP, alkaline phosphatase; γ-GTP, gamma-glutamyl transpeptidase; HbA1c, hemoglobin A1c; CPZ, cefoperazone; ABPC/SBT, ampicillin/sulbactam; PIPC/TAZ, piperacillin/tazobactam Reference ranges (representative values): WBC, 3,300–8,600 /µL; CRP, <0.14 mg/dL; AST, 13–39 IU/L; ALT, 7–52 IU/L; ALP, 38–113 IU/L, γ-GTP: 13-64 IU/L; Blood glucose: 73–109 mg/dL; HbA1c: 4.6–6.2%

Variable	Case 1 (Acute cholecystitis)	Case 2 (Liver abscess)
Age/Sex	70-year-old male	66-year-old male
Comorbidities	Cerebral infarction, atrial fibrillation, type 2 diabetes mellitus, and hypertension	Hypertension, dyslipidemia
Social history	Alcohol (-), smoking (-)	Alcohol (+), smoking (+)
Chief complaints	Fever, vomiting	Fever, general fatigue
Presumed source of infection	Periodontitis	Dental caries
Causative organism	Porphyromonas gingivalis	Actinomyces naeslundii
WBC (/µL)/CRP (mg/dL)	25,100/39.27	13,300/14.25
AST/ALT (IU/L)	55/53	32/45
ALP/γ-GTP (IU/L)	97/62	232/69
Blood glucose (mg/dL)/HbA1c (%)	189 / 6.0	Not available
Imaging findings	Gallbladder enlargement with a hepatic surface abscess	Abscess formation in the left hepatic lobe
Treatment	Drainage + CPZ → ABPC/SBT	Drainage + PIPC/TAZ → ABPC/SBT
Outcome	Discharged on hospital day 28	Discharged on hospital day 33

**Figure 2:**
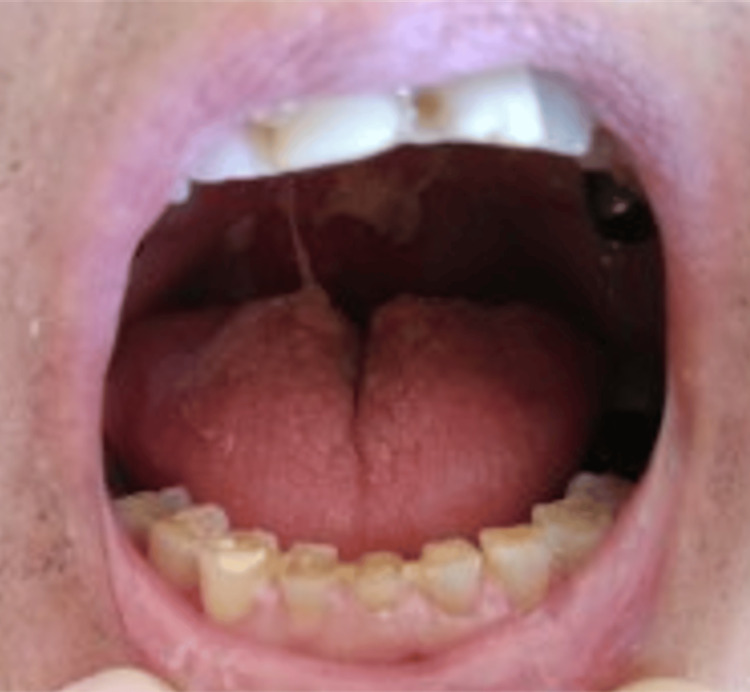
Intraoral findings of Case 1 show periodontitis.

Treatment included intravenous cefoperazone and percutaneous drainage. Bacterial identification was performed using the VITEK 2 Compact system with the Anaerobe and Corynebacterium (ANC) identification card (bioMérieux, Marcy-l'Étoile, France). Before initiating antibiotic therapy, bile culture showed *Porphyromonas gingivalis*, and no other organisms were detected. The isolate showed no susceptibility to cefoperazone; therefore, the treatment was switched to ampicillin-sulbactam, to which the bacterium was confirmed to be susceptible. The modification of the antimicrobial therapy based on the bacterial identification led to an improvement in the patient’s inflammatory markers and hepatobiliary enzymes. CT imaging showed resolution of the cholecystitis. He was discharged on hospital day 28. Intraoral findings of the patient showed periodontitis (Figure 2).

Case 2

The patient was a 66-year-old man who was hospitalized with fever and general fatigue. CT imaging suggested a liver abscess, and antibiotic therapy was initiated. However, the symptoms worsened, and repeat imaging revealed enlargement of the abscess, prompting transfer to our hospital for evaluation and treatment.

The patient’s medical history included hypertension and dyslipidemia. He consumed 14 g of alcohol daily and smoked 20 cigarettes per day from the age of 20 to 50 years. There was no family history of liver disease.

**Figure 3:**
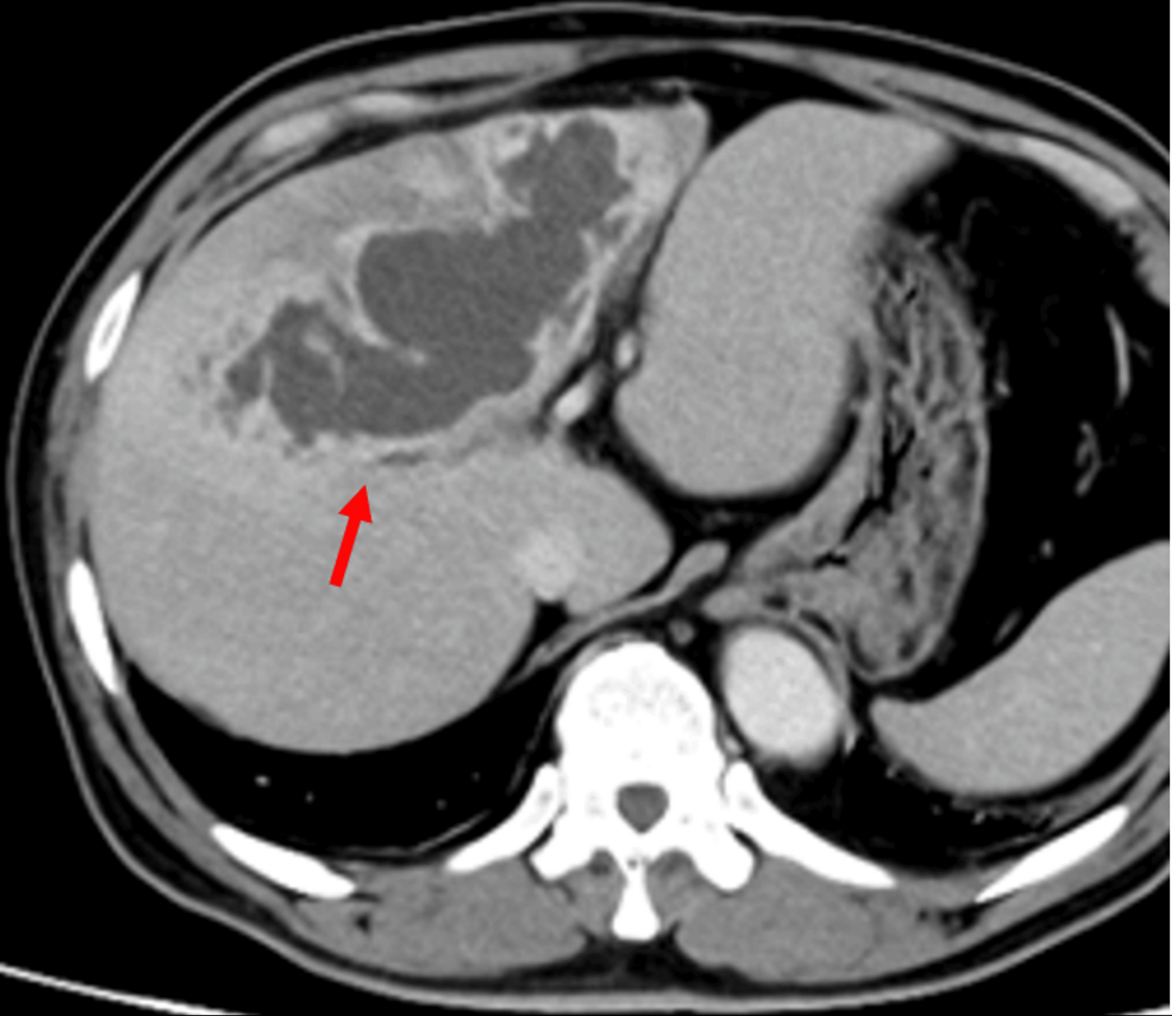
Contrast-enhanced CT shows a liver lesion. The red arrow points to a low-attenuation area in the left lobe of the liver with peripheral ring enhancement.

At admission, the patient was alert and afebrile. Jaundice was not present. Abdominal examination revealed mild tenderness in the epigastric region without rebound tenderness or guarding. Laboratory tests showed leukocytosis and elevations in CRP, AST, ALT, alkaline phosphatase (ALP), and gamma-glutamyl transpeptidase (γ-GTP) (Table 1). Inflammatory markers were significantly increased. Contrast-enhanced CT showed a low-attenuation area in the left lobe of the liver with peripheral ring enhancement (Figure 3). The patient was diagnosed with a liver abscess.

**Figure 4:**
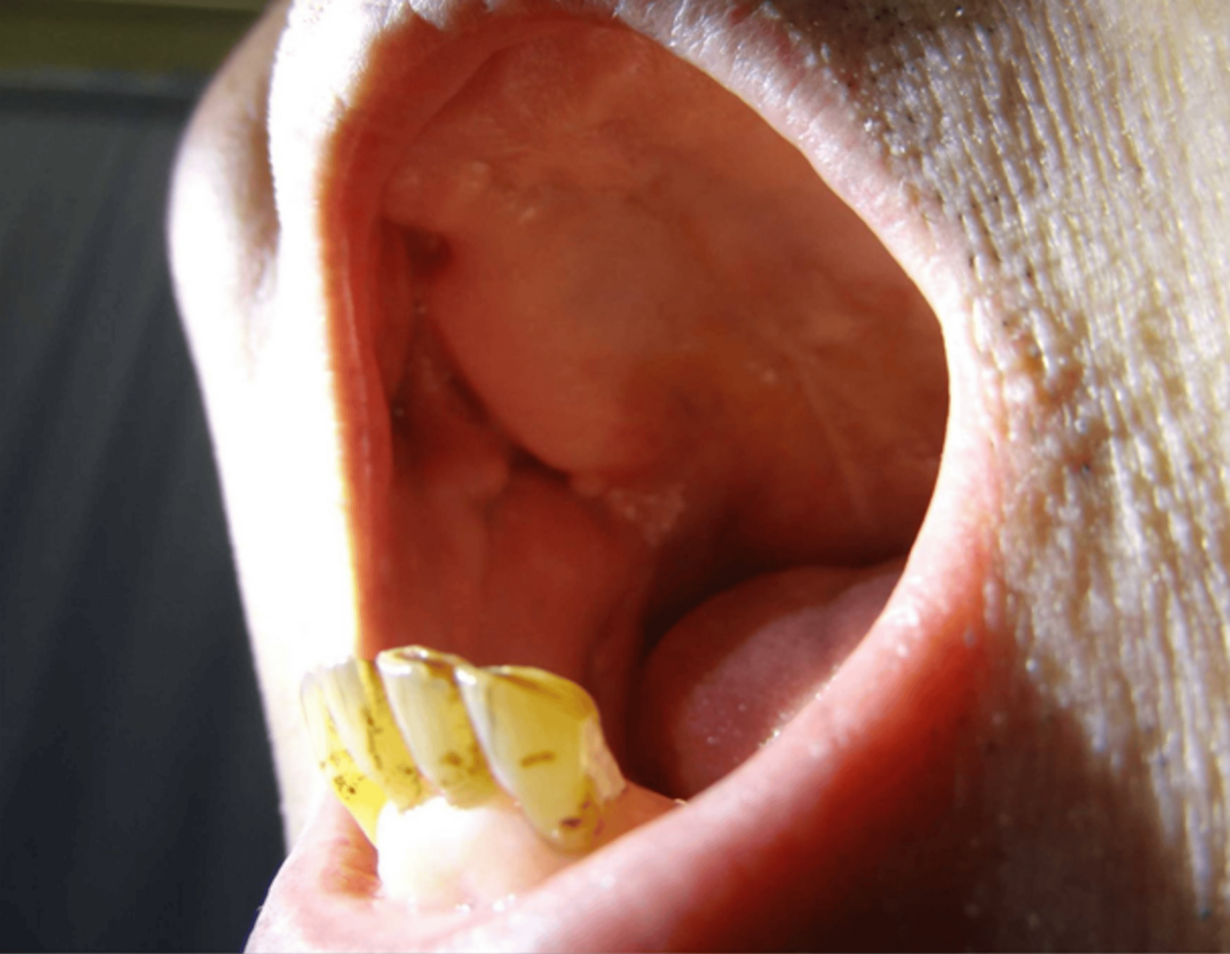
Intraoral findings of Case 2 show multiple dental caries.

The patient was treated with piperacillin-tazobactam and percutaneous drainage. Bacterial identification was performed using the VITEK 2 Compact system with the ANC identification card. Before initiating antibiotic therapy, culture of the abscess showed *Actinomyces* ​​*naeslundii*, and no other organisms were detected. Since the isolate was confirmed to be susceptible to ampicillin-sulbactam, the antibiotic was switched to ampicillin-sulbactam, which provides strong activity against anaerobes. The modification of the antimicrobial therapy based on the bacterial identification led to an improvement in the patient’s inflammatory markers and hepatobiliary enzymes. CT revealed a reduction in the size of the abscess. He was discharged on hospital day 33. Intraoral findings of the patient showed multiple dental caries (Figure 4).

## Discussion

The concept of oral focal infection, first proposed by Willoughby D. Miller in 1891, describes the systemic effects of localized oral infections [4]. Oral microorganisms can reach distant organs via hematogenous, enteric, or immune-cell-mediated routes; poor oral hygiene may exacerbate this dissemination [5]. Oral focal infections have been linked to infective endocarditis, cardiovascular disease, pulmonary infections, preterm birth, low birth weight, diabetes, prosthetic infections, and brain abscesses [1].

In these two cases, although oral cultures were not performed and a definitive causal link cannot be established, an association with oral lesions is considered clinically plausible. This is based on the fact that the isolates are typical oral flora, oral infections were present, and no other focus of infection was identified.

*Porphyromonas gingivalis*, a gram-negative bacillus of the genus *Porphyromonas*, is a pathobiont in the oral microbiome that proliferates in periodontal lesions and causes tissue destruction [6]. In Case 1, the patient had clinical signs of periodontitis and no prior history of biliary tract disease (Figure 2). The bacteria are suspected to have spread to the gallbladder via hematogenous routes, suggesting a potential link to the oral infection.

Although cholecystitis caused by oral bacteria is rare, case reports have implicated *Streptococcus gordonii* [7], *Streptococcus gallolyticus* subspecies pasteurianus [8], *Lactobacillus salivarius* [9], and *Lactobacillus paracasei *[10]. However, a search on the PubMed database using the keywords “*P. gingivalis*” and “cholecystitis” yielded no results. 

*Actinomyces naeslundii*, an anaerobic gram-positive bacillus belonging to the genus *Actinomyces*, is a commensal organism in the oropharynx, gastrointestinal tract, and female genital tract [11,12]. In the oral cavity, it is involved in biofilm formation on the gingiva and has been isolated from the dental plaque of patients with chronic periodontitis [11,12].

In Case 2, the patient had poor oral hygiene with multiple carious teeth (Figure 4). Similar to prior reports of liver abscesses caused by oral commensals [13], the bacterium likely spread from the oral cavity via hematogenous routes in the absence of any gastrointestinal or biliary disease, suggesting a potential link to the oral infection.

Osawa reviewed 11 cases of liver abscesses of dental origin reported between 1987 and 2013, with *Fusobacterium* and *Streptococcus *species being the most frequently identified pathogens [13]. None of these cases involved *Actinomyces *species. Hepatic infections caused by *Actinomyces *are typically described as hepatic actinomycosis, which accounts for only 5% of all actinomycosis cases, making it very rare [11,12]. The most common causative species is *Actinomyces israelii* [11,14]. According to Chegini et al., among 32 reported cases of hepatic actinomycosis, *Actinomyces israelii* accounted for 17 cases, whereas *Actinomyces naeslundii* was identified in only four, underscoring its rarity as a hepatic pathogen [14].

The reported risk factors for liver abscesses include diabetes mellitus and immunosuppression due to malignancy [15]. However, Osawa found that only two of 11 patients with dental-origin hepatic actinomycosis had immunodeficiency, suggesting that dental infections alone can cause liver abscesses even in immunocompetent individuals [13]. Case 2involved a patient without significant immunosuppression, indicating that oral infection itself is a sufficient risk factor.

## Conclusions

Universal access to oral health care is becoming a global public health priority, which further highlights the clinical importance of addressing oral bacteria as a potential source of systemic infections. There has been an increasing global awareness of the importance of oral hygiene in recent years. The cases where two oral-derived bacteria cause hepatobiliary infections are rare, suggesting that poor oral hygiene can lead to systemic infections. These cases highlight the importance of oral hygiene in the prevention of systemic diseases. Strengthening collaboration between the medical and dental fields is essential moving forward.
